# Medial Meniscal Extrusion After Anterior Cruciate Ligament Reconstruction (ACLR) Associated With Meniscal Repair and Preoperative Extrusion

**DOI:** 10.7759/cureus.69987

**Published:** 2024-09-23

**Authors:** Ryu Ito, Shotaro Watanabe, Takuya Sakamoto, Kaoru Toguchi, Manato Horii, Seiji Kimura, Satoshi Yamaguchi, Seiji Ohtori, Takahisa Sasho

**Affiliations:** 1 Center for Preventive Medical Sciences, Chiba University, Chiba, JPN; 2 Department of Orthopedic Surgery, Graduate School of Medicine, Chiba University, Chiba, JPN; 3 Department of Orthopedic Surgery, Narashino Daiichi Hospital, Chiba, JPN; 4 Graduate School of Global and Transdisciplinary Studies, College of Liberal Arts and Sciences, Chiba University, Chiba, JPN

**Keywords:** anterior cruciate ligament reconstruction, medial meniscal extrusion, medial meniscus repair, meniscus tear, ramp lesion

## Abstract

Introduction: The risk of post-traumatic osteoarthritis remains high even after anterior cruciate ligament reconstruction (ACLR). Medial meniscal extrusion (MME) is a valuable clinical sign as an early morphological change. This study aimed to analyze MME before and after ACLR and investigate the factors affecting postoperative MME.

Materials and methods: This study included patients who underwent anatomical double-bundle ACLR between January 2016 and July 2021. MME was measured using MRI preoperatively and one year postoperatively. The medial meniscus (MM) treatments were categorized into three groups: no MM injury and no repair (no injury/no repair (N/N)), MM injury but no repair (injury/no repair (I/N)), and MM injury and repair (injury/repair (I/R)). We investigated the factors influencing MME after ACLR using multiple linear regression analysis and compared MME before and after ACLR using paired t-tests.

Results: This study included 133 patients, of whom 90 (37 males and 53 females) were analyzed. The mean age of the patients at surgery was 27.5 years, and 41, 27, and 22 patients were assigned into N/N, I/N, and I/R groups, respectively. Preoperative MME (p<0.001) and I/R (p<0.001) had significant effects on postoperative MME in a regression analysis. Postoperative MME had greater effects than the preoperative MME in all cases (1.16 and 1.53 mm (p<0.01)) and in every MM treatment group (N/N: 1.02 and 1.32 mm (p<0.01), I/N: 1.16 and 1.44 mm (p<0.01), and I/R: 1.42 and 2.05 mm (p<0.001)).

Conclusions: Larger preoperative MME and receiving MM repair were significantly associated with a larger MME after ACLR. Postoperative MME in ACLR patients was significantly greater than preoperative MME.

## Introduction

Medial meniscal extrusion (MME) is a crucial factor in the development of knee osteoarthritis (OA) [1-3]. As the degree of MME increases, the meniscus' ability to distribute load stress on the knee joint is compromised, leading to increased cartilage loss and bone marrow lesions, ultimately resulting in OA [4-7]. This underlines the importance of MME as a potential predictor of OA, a topic that has garnered significant attention.

Several studies have reported that 50-90% of knees with anterior cruciate ligament (ACL) injuries develop post-traumatic OA (PTOA) owing to joint instability [8,9]. A high risk of PTOA development remains even after ACL reconstruction (ACLR) [8,10]. Furthermore, meniscal injury, another factor that leads to PTOA development, is often observed in ACL injuries [11,12]. Thus, meniscal repair is frequently performed in conjunction with ACLR.

In the evaluation of post-ACLR outcomes, MME was used in recent studies. A previous study reported a significant increase in MME before and after ACLR without meniscal repair [13]. Another study reported that the preoperative MME in ACLR patients with longitudinal tears in the medial meniscus (MM) was significantly greater than that in patients without meniscal injury and that preoperative MME did not improve even after MM repair [14]. Moreover, no significant difference in MME was observed between preoperative and 12-month postoperative evaluations after horizontal or cross-suturing using an all-inside device for radial or oblique tears [15].

The effect of meniscal procedures performed during ACLR on MME is not fully understood, particularly concerning the identification of contributing factors and the assessment of changes over time. We hypothesized that postoperative MME was larger than preoperatively and MM treatments had an effect on MME. This study aimed to analyze the value of MME before and after ACLR and to investigate whether MM injury and repairs influence post-ACLR MME. We believe that when evaluating ACLR using MME, we need to consider the influence of MM repairs and understand that the extent of this influence is crucial for future clinical studies.

This article was previously posted to the Research Square preprint server on November 21, 2023 [16].

## Materials and methods

This retrospective observational study was conducted at a single hospital. Medical records were collected anonymously and reviewed retrospectively. Individual informed consent was not obtained because of the anonymous nature of the data and the fact that no new intervention was performed for data collection. The research protocol was approved by the Research Ethics Committee of the Graduate School of Medicine, Chiba University (approval number: M10436, dated September 2022). Data collection was completed by October 2022.

Participants

This study included patients who suffered an ACL rupture and who underwent anatomic double-bundle ACLR between January 2016 and July 2021 and were followed up for one year postoperatively. Patients with multiple ligament injuries, patients who proceeded to the secondary surgery, and patients who did not undergo preoperative or postoperative MRI were excluded.

Data collection

Medical records were reviewed to collect information on the patient’s age, sex, BMI, and time from injury to surgery. Surgical records were reviewed to collect details of the ACLR procedure, site of meniscal injury, meniscal injury pattern, and methods used to treat any meniscal injury. X-rays were used to measure the femorotibial angle (FTA) to evaluate lower limb alignment, and MRI was used to assess the MME.

Surgical procedure

Anteromedial and anterolateral portals were created, and a 30° arthroscope was used to confirm the presence of meniscal injury. The type of meniscal procedure (repair or meniscectomy) was determined according to the type of injury. Anatomical ACLR was performed using hamstring tendon autografts, and femoral and tibial tunnels were created using the outside-in technique. An ACL TightRope^TM^ (Arthrex, Naples, FL, USA) or EndoButton CL^TM^ (Smith & Nephew, Andover, MA, USA) was used for femoral graft fixation, whereas TensionLoc^TM^ (Arthrex) or ACL TightRope ABS^TM^ (Arthrex) was used for tibial graft fixation. During graft fixation, the anteromedial bundle was fixed at 20° of knee flexion with 40 N traction, whereas the posterolateral bundle was fixed in knee extension with 40 N traction.

Treatment of meniscal injuries

Knees without MM injuries were assigned to the no injury/no repair (N/N) group. Patients with MM injury in the red-white or red zone without instability upon probing, for which repair was not performed, or those with a small flap injury in the white zone, for which partial meniscectomy was performed, were assigned to the injury/no repair (I/N) group. Patients with MM injury and instability who underwent repair were assigned to the injury/repair (I/R) group. Repairs were performed using all-inside, outside-in, and inside-out sutures, with the majority being all-inside sutures.

Postoperative treatment

Patients who underwent meniscal repair had a range of motion limited to 90° of knee joint flexion for up to four weeks after the operation, with no range of motion restrictions after four weeks. However, in cases where the meniscus was left untreated or meniscectomy was performed, the range of motion was not restricted. Patients were advised to undergo half weight-bearing for two weeks after the operation, two-thirds weight-bearing for two to three weeks postoperatively, and complete weight-bearing was initiated three weeks postoperatively.

Measurement of MME

**Figure 1:**
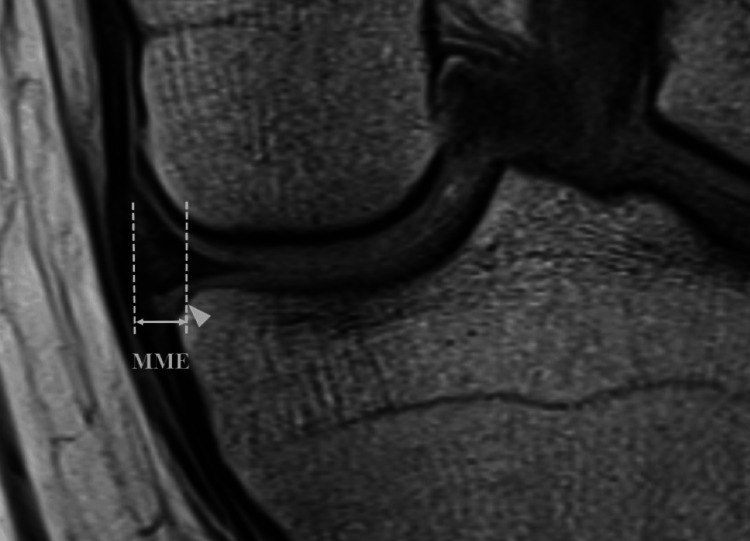
Measurement of MME using MRI The MME was determined by measuring the distance from the innermost edge of the MM to the medial border of the tibia, excluding the osteophyte, in the coronal slice where the intercondylar ridge was the greatest. The arrowhead indicates the medial border of the tibia, excluding the osteophyte. It marks the location of the inflection point where the horizontal line of the joint surface drops into a curved depression. MME: medial meniscal extrusion, MRI: magnetic resonance imaging, MM: medial meniscus

Retrospective measurements of MME using pre- and postoperative MRI were conducted by an experienced orthopedic surgeon who had performed orthopedic surgeries for >5 years. Retrospective measurements of MME using pre- and postoperative MRI were conducted by an experienced orthopedic surgeon who had performed orthopedic surgeries for >5 years. Preoperative MME was measured at the time of injury, and postoperative MME was measured one year postoperatively using MRI. MRI was performed using a 3.0 Tesla MRI scanner (General Electric, Milwaukee, WI, USA) with the following imaging parameters: TR/TE, 2,000/30 ms; matrix, 416 × 416; field of view, 14 cm; and slice thickness, 3 mm. The sequence used in this study was proton density-weighted coronal imaging. Some images were obtained using different imaging protocols by the referring physicians, especially at the time of injury. However, the images were scanned with the same proton density-weighted, slick thickness, and resolution. Preoperative and postoperative MME were determined by measuring the distance from the innermost edge of the MM to the medial border of the tibial articular surface, excluding the osteophyte, in the coronal slice, where the intercondylar ridge was the greatest (Figure 1) [17].

To evaluate the reproducibility of the measurements, two orthopedic surgeons measured the same 20 patients and remeasured them at intervals of >2 months.

Additional clarification of medial meniscal injury

MM injury patterns were classified into seven categories: ramp tear, complex tear, radial tear, bucket handle tear, medial meniscal posterior root tear, longitudinal tear, and others. A ramp lesion is defined as a meniscocapsular tear of the posterior MM observed in the trans-notch view during arthroscopy at the time of surgery. In addition, the ramp lesions with instability on probing were repaired, and no surgery was performed if the lesion was stable. The MM injury locations were classified into two categories: the medial segment and the other segment. The medial segment is defined as the central part of the meniscus when it is divided into three equal sections in the anterior-posterior direction.

Statistical analysis

All statistical analyses were performed using EZR (Saitama Medical Center, Jichi Medical University, Saitama, Japan), a graphical user interface for R. Statistical significance was set at p<0.05. The reproducibility of the MME measurements was verified by examining the intraclass correlation coefficients (ICC) (1,2), ICC (2,1), and ICC (3,1).

Primary analyses identified the factors influencing MME after ACLR and compared the MME before and after the ACLR. After confirming normality with histograms, multiple linear regression analysis was performed with postoperative MME as the dependent variable and age, sex, BMI, preoperative MME, and MM treatments as independent variables. The MM treatments were classified into three groups: N/N, I/N, and I/R. Age was measured in years, BMI in kg/m^2^, and preoperative MME in millimeters. Sex was coded as 1 = male or 0 = female; I/N as 1 = yes or 0 = no; or I/R as 1 = yes or 0 = no. We compared the MME before and after the ACLR using a paired t-test and compared in the N/N, I/N, and I/R groups.

As exploration analyses, using the Kruskal-Wallis test, we compared the preoperative and postoperative MME between the N/N, stable ramp lesion, and unstable ramp lesion groups. Post-hoc tests were conducted using Bonferroni’s multiple comparison test. Using the Mann-Whitney U test, we also compared the preoperative and postoperative MME between MM injuries in the medial and other segments. We examined the correlation between FTA and MME using Pearson's correlation.

## Results

**Figure 2:**
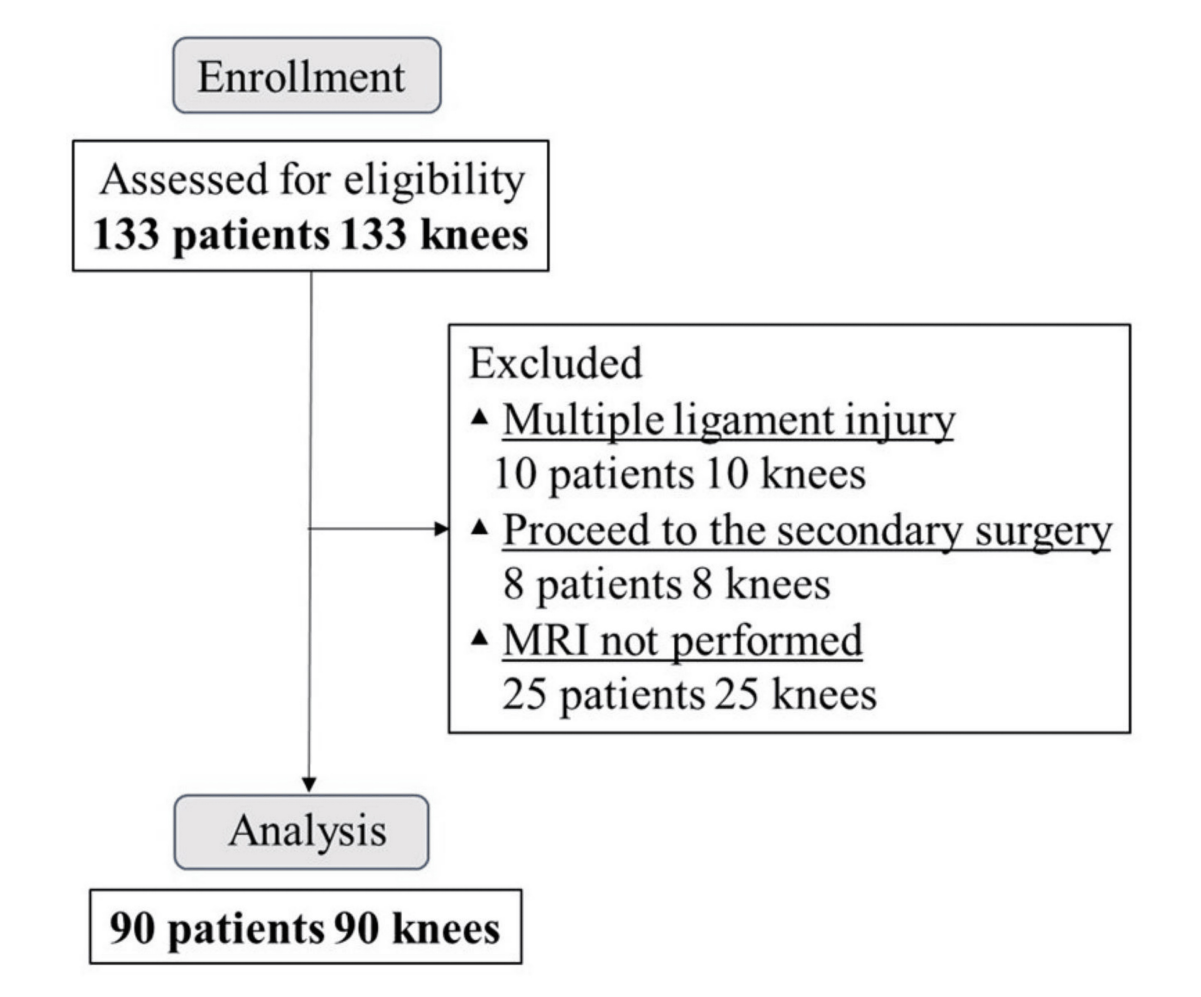
Flow of participants in the study MRI: magnetic resonance imaging

This study included 133 knees from 133 patients. However, 10 patients with complex ligament injuries, eight with reoperation, and 25 without preoperative or postoperative MRI were excluded from the analysis. As a result, 90 knees from 90 patients were analyzed (Figure 2).

Thirty-seven male and 53 female patients were included in the study. The mean age at the time of surgery was 27.5 years. The median time between the injury and surgery was 3.5 months. Forty-one, 27, and 22 patients were assigned to the N/N, I/N, and I/R groups, respectively (Table 1).

**Table 1:** Demographics of the participants * data are expressed as counts (%), ** data expressed as mean ± standard deviation, *** data representing the period from injury to surgery expressed in months as the median n: number of knees, BMI: body mass index, N/N: no MM injury and no repair, I/N: MM injury but no repair, I/R: MM injury and repair, MM: medial meniscus

Characteristics (n=90)			N/N (n=41)^*^	I/N (n=27)^*^	I/R (n=22)^*^
Sex^*^	Male	37 (41%)	17 (41%)	11 (41%)	9 (41%)
	Female	53 (59%)	24 (59%)	16 (59%)	13 (59%)
Age (years)^**^		27.5 ± 11.4	25.6 ± 10.3	30.5 ± 11.4	28.2 ± 12.9
BMI (kg/m²)^**^		23.0 ± 3.63	22.7 ± 3.1	22.7 ± 3.2	23.7 ± 4.7
Time from injury to surgery (months)^***^		3.5 (1-300)	3.0	5.0	3.0

Out of the 27 cases in the I/N group, partial meniscectomy was performed in six cases.

Reproducibility of MME measurements

Intraclass correlation coefficients (1,2) were 0.82, ICC (2,1) were 0.80, and ICC (3,1) were 0.89, indicating good reliability.

Factors affecting postoperative MME

Preoperative MME (p<0.001) and receiving MM repair (p<0.001) were significant predictors of postoperative MME (Table 2).

**Table 2:** Significant factors affecting postoperative MME MM treatments were classified into three groups: N/N, I/N, and I/R. Sex was coded as 1 = male or 0 = female; I/N as 1 = yes or 0 = no; or I/R as 1 = yes or 0 = no. * significant difference BMI: body mass index, MME: medial meniscal extrusion, I/N: MM injury but no repair, I/R: MM injury and repair, CI: confidence interval, MM: medial meniscus

	Parameter estimate (B)	Lower limit 95% CI of (B)	Upper limit 95% CI of (B)	p-value
Age (years)	0.002	-0.005	0.010	0.49
BMI (kg/m^2^)	-0.017	-0.043	0.009	0.20
Preoperative MME (mm)	0.701	0.579	0.823	<0.001^*^
Sex	-0.019	-0.207	0.168	0.838
I/N	0.016	-0.186	0.218	0.876
I/R	0.466	0.245	0.687	<0.001^*^

The absence of multicollinearity was confirmed by a variance inflation factor of <1.3 among the independent variables. Additionally, the normality of the residuals was confirmed. The regression equation was significant (p<0.001), with an R² value of 0.69.

Comparison of MME before and after ACLR

The preoperative and postoperative MME were 1.16 ± 0.75 and 1.53 ± 0.73 mm, respectively, revealing significant differences (p<0.01). In the N/N, I/N, and I/R groups, the postoperative MME were significantly greater than the preoperative MME (Table 3).

**Table 3:** Comparison of MME before and after ACLR MME: medial meniscal extrusion, ACLR: anterior cruciate ligament reconstruction, SD: standard deviation, N/N: no MM injury and no repair, I/N: MM injury but no repair, I/R: MM injury and repair, MM: medial meniscus * significant difference

	n	Preoperative (mm) (mean MME ± SD)	Postoperative (mm) (mean MME ± SD)	p-value
All cases	90	1.16 ± 0.75	1.53 ± 0.73	<0.01^*^
N/N	41	1.02 ± 0.62	1.32 ± 0.45	<0.01^*^
I/N	27	1.16 ± 0.87	1.44 ± 0.77	<0.01^*^
I/R	22	1.42 ± 0.80	2.05 ± 0.85	<0.01^*^

Among cases with meniscal injuries, we focused on the more frequent Ramp lesions and compared preoperative and postoperative MME between the N/N, I/N, and I/R groups. I/N was defined as stable ramp lesions and I/R as unstable ramp lesions. Comparing the MME between the two and N/N groups, there were no significant differences preoperatively, but there were significant differences postoperatively (unstable ramp and N/N (p<0.01); unstable ramp and stable ramp (p<0.01)) (Table 4).

**Table 4:** Comparison of preoperative and postoperative MME between the N/N, I/N, and I/R groups, focusing on ramp lesions * in the post-hoc test, Bonferroni's multiple comparison test revealed significant differences between unstable ramp lesions and N/N (p<0.01) and between unstable ramp lesions and stable ramp lesions (p<0.01). n: number of knees, N/N: no MM injury and no repair, I/N: MM injury but no repair, I/R: MM injury and repair, MME: medial meniscal extrusion, ACLR: anterior cruciate ligament reconstruction, MM: medial meniscus

	N/N (n=41)	I/N (stable ramp lesion) (n=18)	I/R (unstable ramp lesion) (n=7)	p-value
Preoperative (mm)	1.02 ± 0.62	1.13 ± 0.50	1.39 ± 0.96	0.7
Postoperative (mm)	1.32 ± 0.45	1.41 ± 0.49	2.17 ± 1.00	<0.01*

The MM injury patterns are presented in Table 5.

**Table 5:** Classification based on the type of MM injuries Types of MM injuries were classified into six categories. The "other" category includes small tears and flap tears. n: number of knees, MMPRT: medial meniscal posterior root tear, MM: medial meniscus

Meniscus tear pattern	n	Preoperative (mm)	Postoperative (mm)
Ramp tear	25	1.20 ± 0.65	1.62 ± 0.74
Complex tear	6	2.15 ± 1.27	2.52 ± 1.22
Radial tear	1	1.5	1.7
Bucket handle tear	4	1.08 ± 0.81	1.43 ± 0.98
MMPRT	1	1.5	1.6
Longitudinal tear	7	1.06 ± 0.67	1.57 ± 0.64
The other	5	2.05± 1.51	2.05± 0.98

We also investigated the sites of meniscal injuries assigned to the medial and other segment groups, and no significant difference was observed between the two groups (Table 6).

**Table 6:** Classification based on MM injury location The medial segment refers to the central third of the MM. n: number of knees, MM: medial meniscus

Tear of MM location	Medial segment (n=15)	The other segment (n=34)	p-value
Preoperative (mm)	1.57 ± 1.26	1.10 ± 0.53	0.237
Postoperative (mm)	2.05 ± 1.12	1.57 ± 0.70	0.101

There was no correlation between FTA and preoperative MME (correlation coefficient: 0.18, p=0.09) nor between FTA and postoperative MME (correlation coefficient: 0.07, p=0.51).

## Discussion

The most important findings of the present study were that preoperative MME and receiving MM repair significantly influenced postoperative MME, and postoperative MME was significantly greater than preoperative MME (p<0.01).

We observed that the postoperative MME remained larger in patients who underwent MM repair. This finding is consistent with a previous report that indicated no MME improvement after suturing of longitudinal MM tears in ACLR, suggesting that preoperative MME does not improve even with the repair of MM tears [14]. Another study reported that suturing with an all-inside suture device in patients with radial or oblique posterior MM tears did not improve MME [15]. This study is different from ours because it focused on patients with MM injuries who did not undergo ACLR. Nevertheless, the finding that MME did not improve with repair is consistent with the findings of our study.

We observed that postoperative MME was greater in patients who underwent MM repair. A previous study demonstrated that larger displacements (>3 mm) are frequently associated with radial or posterior root tears [18-20], indicating that a larger preoperative MME may be indicative of these types of injuries. However, although our cases included few such injuries, we found that receiving MM repair was an independent factor associated with increased postoperative MME. We believe larger MM injuries, which require repair, may impact postoperative MME. However, the possibility that suturing itself influenced MME should also be considered, and further research is needed. In this study, we also assigned the sites of meniscal injuries to the medial segment and the other segment. However, there was no significant difference in preoperative or postoperative MME, suggesting that the sites of meniscal injuries do not affect MME. In this study, we found that an unstable ramp lesion, even if repaired, is a risk factor for larger MME postoperatively. However, we defined unstable ramp lesions as those that were repaired. Therefore, the suture for the repair itself might affect the degree of MME. Previous studies have shown that meniscectomy is a risk factor for OA progression [21-24]. Meniscal repair is expected to delay OA progression more than meniscectomy [12,25]. However, whether meniscal repair delays OA progression compared to meniscectomy has not yet been clarified. The results of the present study suggest that receiving MM repair is a risk factor for a larger MME. However, since meniscal repair leaves more meniscal tissue than meniscectomy, and if the preservation of meniscal tissue is a more critical factor than the MME value for OA development, some benefits may be expected. This should be explored in future high-quality studies.

Narazaki et al. reported that MME increased from 1.2 mm preoperatively to 1.8 mm at an average of 11 months postoperatively in patients with ACLR without meniscal injury [13]. The study excluded the effect of meniscal injury on postoperative MME. Although our study included patients with meniscal injury, significant differences were found in any of the three groups in preoperative and postoperative MME. This result suggests that ACL injury itself may be a risk factor for MME, and ACLR may not stop MME progression. Mariani et al. reported a potential involvement of meniscotibial ligament (MTL) injury in MME, and Hada et al. also reported a possibility that MTL damage during ACL injury may induce osteophyte formation and increase MME [26-30].

This study has limitations. First, this study had a relatively small sample size, although it was considered sufficient to examine the factors that typically affect MME. Second, the follow-up period was short. Third, the timing of preoperative MRI scans was not standardized. Fourth, there was no unified MRI protocol for all images, particularly those obtained prior to surgery. Patients typically bring a referral letter along with an MRI of the knee at the initial visit, and the protocols vary across imaging facilities. However, given that all images included proton density-weighted coronal images with 3-4 mm slice spacing and sufficient resolution, we do not believe this had a significant impact on the results. Finally, the influence of ACLR techniques, such as bone tunnel location and graft type, were not considered. However, the uniformity of the devices used in this study minimized the effects of variations in ACLR procedures performed at a single institution.

## Conclusions

Larger preoperative MME and receiving MM repair were significantly associated with larger MME after ACLR. When assessing MME after ACLR, it's important to consider the effect of a larger preoperative MME and MM repair. Postoperative MME in ACLR patients was significantly greater than preoperative MME.

Further research is required to clarify the relationship between changes in MME and the onset of PTOA. This will contribute to the development of new treatment strategies that can be used to prevent and manage PTOA.
